# Surface and Protein Adsorption Properties of 316L Stainless Steel Modified with Polycaprolactone Film

**DOI:** 10.3390/polym9100545

**Published:** 2017-10-23

**Authors:** Shih-Hang Chang, Yuan-Chien Hsiao

**Affiliations:** Department of Chemical and Materials Engineering, National I-Lan University, I-Lan 260, Taiwan; r0523015@ms.niu.edu.tw

**Keywords:** biomaterials, polycaprolactone, surface modification

## Abstract

The surface and protein adsorption properties of 316L stainless steel (316L SS) modified with polycaprolactone (PCL) films are systematically investigated. The wettability of the PCL films was comparable to that of bare 316L SS because the rough surface morphology of the PCL films counteracts their hydrophobicity. Surface modification with PCL film significantly improves the corrosion resistance of the 316L SS because PCL is insulating in nature. A coating of PCL film effectively reduces the amount of adhered bovine serum albumin (BSA) on the surface of 316L SS in a bicinchoninic acid protein assay. PCL is both biodegradable and biocompatible, suggesting the potential for the surface modification of implants used in human bodies; in these applications, excellent corrosion resistance and anticoagulant properties are necessary.

## 1. Introduction

316L stainless steels (316L SS) are often applied as biomedical metallic materials—including in joint replacements, stents, and orthopedics—because they offer the unique advantages of excellent corrosion resistance, low cost, and good biocompatibility [1,2,3]. However, 316L SS risks the release of Ni^2+^, Cr^3+^, and Fe^3+^ to the body by the corrosion of the Cr oxide surface layer when serving as an implant material [4,5,6]. These metal ions are potential health hazards that may induce allergies and cancer [7,8]. To address this issue, many studies have demonstrated that the corrosion resistance of 316L SS can be improved by coating with various protective films [9,10,11,12,13,14,15,16]. Zhang et al. [9] reported that 316L SS coated with Zr and ZrO_2_ alloyed layers showed enhanced biocompatibility and wear resistance. Al-Rashidy [10] showed that the biocompatibility and corrosion resistance of 316L SS could be improved by adding a bioactive borate glass layer coating using an electrophoretic deposition technique. Sun et al. [11] demonstrated that coating 316L SS with superhydrophobic TiO_2_ nanotube arrays could reduce platelet adhesion and improve corrosion resistance. Sharifnabi et al. [12] deposited Mg-substituted fluoroapatite coatings on the surface of 316L SS using a sol-gel dip coating method to improve the corrosion resistance and biocompatibility of 316L SS implants. Ding et al. [13] showed that a 316L SS surface modified with zirconium carbonitride showed improved blood compatibility and corrosion resistance. Chang et al. [14,15] proposed that CuAlO_2_ and Cu–Al–Ca–O thin films of only 100 nm in thickness could be used to coat 316L SS because they show high surface nanohardness values and excellent corrosion resistances. They also reported that 316L SS coated with an ethylene vinyl acetate/chitosan composite film exhibited a smooth and hydrophobic surface with a low capacity for protein adsorption [16].

Poly(ε-caprolactone) (PCL) is a biocompatible and bioresorbable polymer with potential as a biomaterial for bone tissue engineering, drug delivery, cartilage repair, wound dressing, cardiovascular tissue engineering, and nerve regeneration [17,18,19,20]. However, very few studies have investigated the surface modification applications of PCL for 316L SS. Recently, Kharaziha et al. [21] demonstrated a novel (PCL)-forsterite nanocomposite film deposited on 316L SS using a dip-coating technique to improve the bioactivity and corrosion resistance of the alloy. According to the study, the nanocomposite PCL-forsterite coating rendered 316L SS a bioactive and corrosion-resistant material suitable as a bone substitute. PCL is also non-conducive to cell attachment [18], suggesting that PCL film has potential for biomaterial applications requiring good blood anticoagulant properties. Therefore, the aim of this study is to investigate the surface and protein adsorption properties of 316L SS coated with PCL film.

## 2. Materials and Methods

The 316L SS used in this study was purchased from Soonglee Metals Inc., Kaohsiung, Taiwan, and cut into several specimens measuring 20 mm × 30 mm × 1 mm using a low-speed diamond saw (Buehler IsoMet, Chicago, IL, USA). The surface of the 361L SS was ground with abrasive papers and then polished using 0.3 μm Al_2_O_3_ powder. The PCL powders (Model: CAPA6800), with a molecular weight of approximately 80,000 g mol^−1^, were purchased from Widescope Ltd., Taipei, Taiwan. Individually, 0.1, 0.2, 0.3, 0.4, and 0.5 g of PCL powder was mixed with 10 mL of acetone in a beaker covered by aluminum foil to obtain PCL solutions of various concentrations. Each solution was stirred at 25 °C for 60 min and then poured onto the surface of the 316L SS specimen to obtain a PCL film. Then, each PCL film-coated specimen was dried at 45 °C in an oven for 24 h to obtain a dense PCL film. The specimens coated with 0, 0.1, 0.2, 0.3, 0.4, and 0.5 g PCL powders are designated as bare 316L SS, 1% PCL, 2% PCL, 3% PCL, 4% PCL, and 5% PCL, respectively, in the following report. However, it was difficult to obtain a dense film with 0.1 g of PCL powders, and the surface of the PCL became very rough for the 0.5 g PCL powder specimen. Therefore, only the bare 316L SS, 2% PCL, 3% PCL, and 4% PCL specimens were subjected to the following characterization tests.

The functional groups of the PCL films were detected using a Spectrum 100 (PerkinElmer, Waltham, MA, USA) attenuated total reflectance Fourier transform infrared (ATR-FTIR) spectrometer. Each specimen was measured in the 4000–400 cm^−1^ frequency range, with 16 scans run at a resolution of 4 cm^−1^. The wettability properties of the surfaces of the PCL films were measured using a FTA125 (First Ten Ångstroms, Portsmouth, OH, USA) contact-angle instrument. The surface morphologies of the PCL films were observed using a Tescan 5136MM scanning electron microscope (SEM) (Tescan, Kohoutovice, Czech Republic). The cathodic and anodic polarization Tafel curves of each sample were determined using an ECW-5600 (Jiehan, Taichung, Taiwan) electrochemical workstation. The protein adsorption properties of the PCL films were analyzed by performing a bicinchoninic acid (BCA) protein assay using bovine serum albumin (BSA). The BSA and BCA reagents were both purchased from Bio Basic Inc., Toronto, ON, Canada. The phosphate buffer solution (PBS) and sodium dodecyl sulfate (SDS) used in the BCA protein assay were purchased from UniRegion (Taichung, Taiwan) and Sigma-Aldrich (St. Louis, MO, USA), respectively. The optical density (OD) values of the specimens were determined at 562 nm in a GENESYS 20 (Thermo Scientific, Waltham, MA, USA) spectrophotometer.

## 3. Results

**Figure 1:**
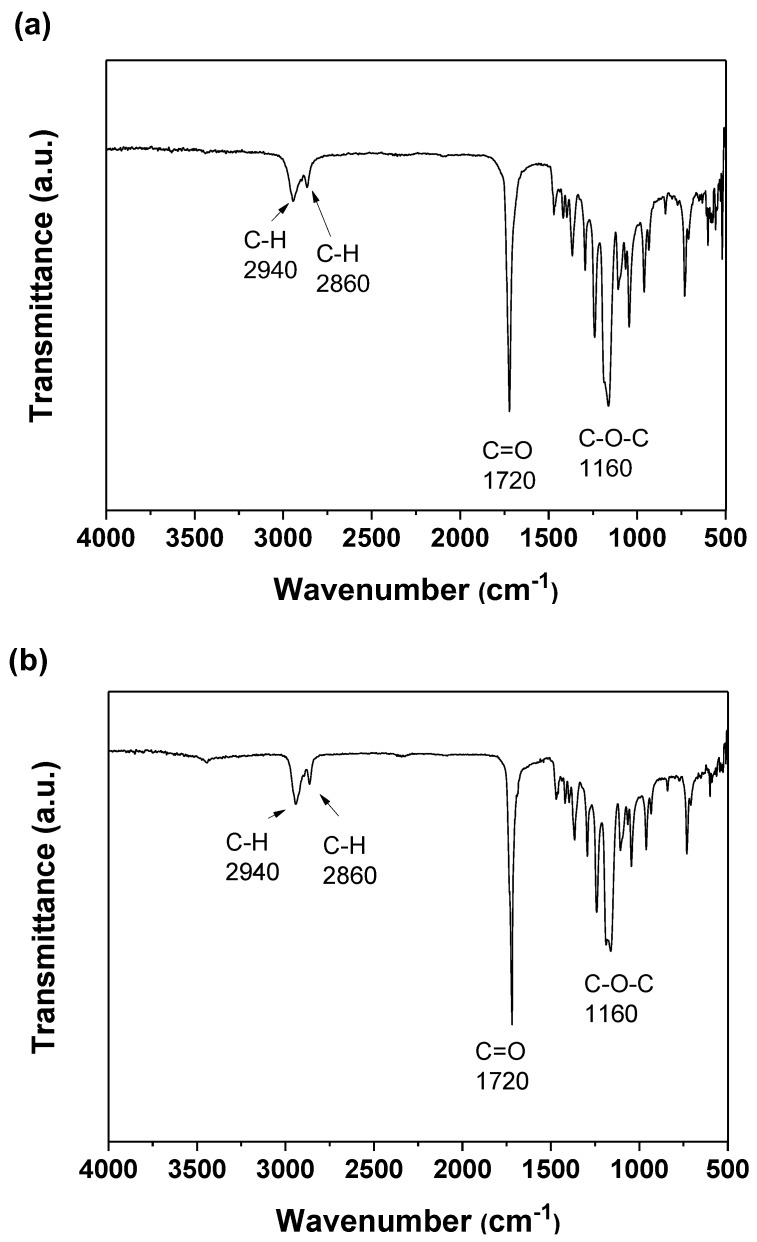
The ATR-FTIR spectra of the (**a**) 316L SS coated with a 2% PCL film and (**b**) 2% PCL film without coated on 316L ss.

Figure 1a,b show the ATR-FTIR spectra of the 316L SS coated with the 2% PCL film and the 2% PCL film without coating on 316L SS, respectively. Figure 1 reveals that the spectra of the 2% PCL film with or without coating on 316L SS are almost identical. Each spectrum exhibits a C–O–C ether group stretching absorption band at approximately 1160 cm^−1^ and a C=O carbonyl group stretching absorption band at approximately 1720 cm^−1^. Figure 1 also shows C–H stretching absorption bands at approximately 2860 and 2940 cm^−1^. The ATR-FTIR spectra of the 316L SS coated with 3% and 4% PCL films are not presented here because they are very similar to that of the 2% PCL film presented in Figure 1.

**Figure 2:**
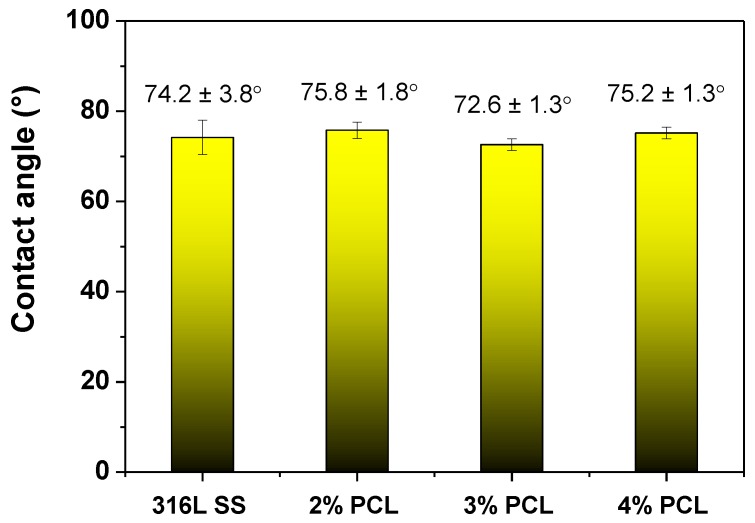
The water contact angles of the bare 316L SS and the 316L SS coated with 2, 3, and 4% PCL films.

Figure 2 plots the water contact angle measurement results of the bare 316L SS and the 316L SS coated with 2, 3, and 4% PCL films. According to Figure 2, the bare 316L SS exhibits a contact angle value of 74.2 ± 3.8°, whereas those of the 2, 3, and 4% PCL films are determined as 75.8 ± 1.87°, 72.6 ± 1.3°, and 75.2 ± 1.3°, respectively. The wettability properties of the bare 316L SS and that coated with PCL films of various concentrations are not significantly different.

**Figure 3:**
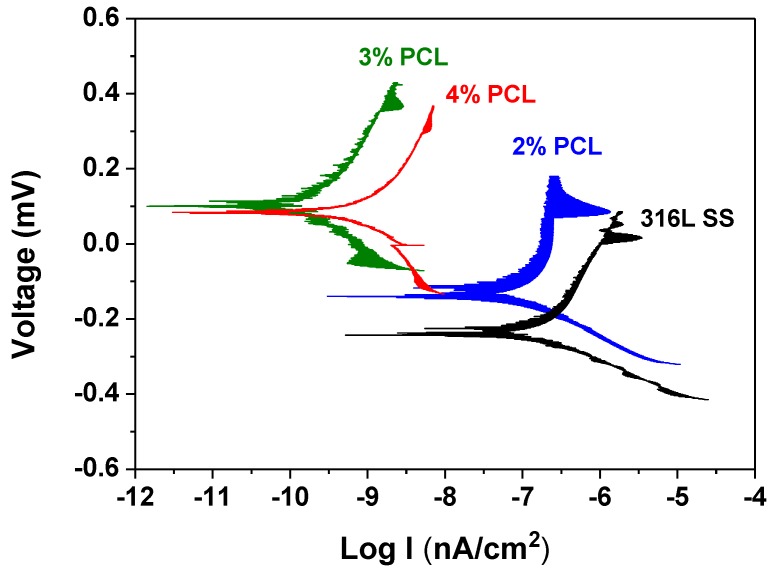
The cathodic and anodic polarization Tafel curves of the bare 316L SS and the 316L SS coated with 2, 3, and 4% PCL films.

Figure 3 shows the selected cathodic and anodic polarization Tafel curves determined in Ringer’s solution at 37 °C from the bare 316L SS and the 316L SS coated with 2, 3, and 4% PCL films. Table 1 lists the determined average corrosion potential (*E*_corr_) and corrosion current density (*i*_corr_) values of each specimen. From Table 1, the *E*_corr_ value of the bare 316L SS is −196.2 mV, whereas those of the 316L SS coated with 2, 3, and 4% PCL films are −129.6, 96.0, and 84.9 mV, respectively. The corrosion potential is the characteristic of material’s surfaces to lose electrons in the presence of an electrolyte. This indicates that the corrosion resistance of the 316L SS is significantly improved by coating with the PCL film when immersed in Ringer’s solution. Table 1 also shows the *i*_corr_ value of the bare 316L SS of 97.5 nA/cm^2^. Meanwhile, the *i*_corr_ values of the 2, 3, and 4% PCL films are 84.7, 0.1, and 0.3 nA/cm^2^, respectively. Since corrosion current density is proportional to the corrosion rate of the material. This demonstrates that the corrosion rate of the 316L SS in Ringer’s solution is effectively decreased by the deposition of the PCL films.

**Figure 4:**
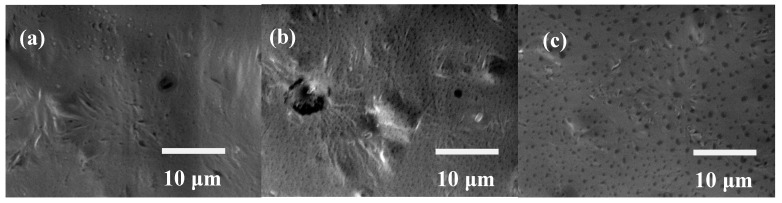
SEM images of the 316L SS coated with (**a**) 2%; (**b**) 3%; and (**c**) 4% PCL films.

Figure 4a–c show the SEM images of the 316L SS coated with 2, 3, and 4% PCL films, respectively. Figure 4a shows that the surface morphology of the 2% PCL film is somewhat rough. In addition, some holes are observed on the surface of the 2% PCL film. Compared to the SEM image of the 2% PCL film, Figure 4b shows that the surface of the 3% PCL film is rougher with more abundant holes. Some significant cavities are also visible on the surface of the 3% PCL film. Figure 4c shows many holes spread over the surface of the 4% PCL film.

**Figure 5:**
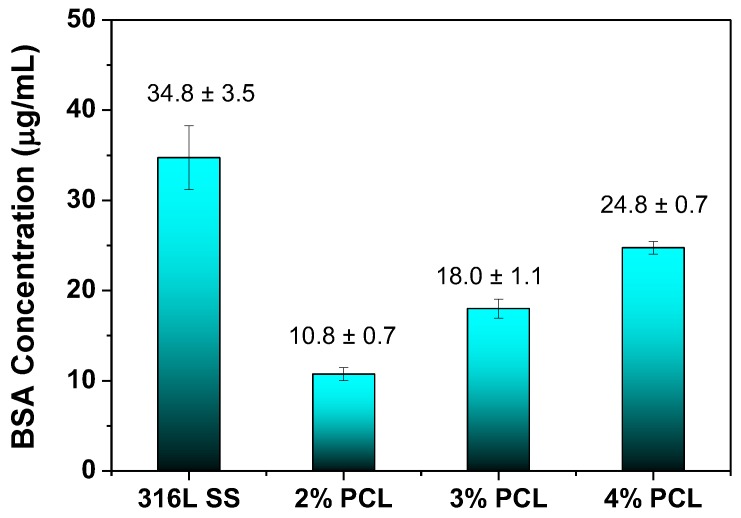
The BSA adhesion concentrations for the bare 316L SS and 316L SS coated with 2, 3, and 4% PCL films, determined from the BCA protein assay.

Figure 5 plots the BSA adhesion concentrations for the bare 316L SS, and 2, 3, and 4% PCL films determined from the BCA protein assay. In the BCA protein assay, each specimen was rinsed three times with PBS followed by immersion in 5 mL of BSA solution at 37 °C for 24 h. Thereafter, each specimen was immersed in 2 mL of a SDS solution for another 24 h; after, 0.1 mL of each solution was mixed with 1 mL of the BSA solution in a cuvette. The BSA concentration was calculated by determining the OD value of each BSA solution in the cuvette using a BSA concentration standard curve. Figure 5 shows that the bare 316L SS exhibits the highest BSA adhesion concentration of 37.3 μg/mL. On the contrary, the 316L SS coated with 2% PCL film exhibits the lowest BSA adhesion concentration of 10.3 μg/mL. In addition, the BSA adhesion concentrations of 316L SS coated with 3% (17.3 μg/mL) and 4% PCL (25.3 μg/mL) films are lower than that of the bare 316L SS. This suggests that the amount of BSA protein adhered to 316L SS can be effectively reduced by coating with PCL film. However, the protein adsorption properties of the 3% and 4% PCL films are less good than that of the 2% PCL film.

## 4. Discussion

PCL films typically exhibit a hydrophobic nature and are non-conducive to cell attachment [18]. However, the wettability measurement results shown in Figure 2 indicate that the determined water contact angle of the bare 316L SS is comparable to those of specimens coated with PCL films. This unexpected result is caused by the rough surfaces of the PCL films, as demonstrated in Figure 4. Meanwhile, several studies have demonstrated that BSA proteins are easily adhered on the surface of stainless steel by physisorption and irreversible chemisorption [22,23,24,25,26]. The surface complexation of proteins and the surface metal hydroxide may cause the corrosion and undesirable metal ions to leach from stainless steel [25,26]. The BCA protein assay results shown in Figure 5 reveal that the amount of the BSA protein adhered to the 316L SS is much higher than that on the 316L SS coated with PCL films, indicating that the PCL films exhibit superior anticoagulant properties compared to those of bare 316L SS, despite the rough surface morphology of the PCL. This characteristic is crucial for the application of 316L SS as an implant material, especially as a blood-contacting biomedical device. In addition, the BSA protein adsorption of the PCL films is slightly increased from the 2% to 4% PCL films. This is because the higher-concentration PCL films show rougher surfaces with more abundant pores. Besides, the strong hydrogen bonding between the carbonyl functional groups of the PCL films and the carboxylic acid of the BSA are also responsible for higher BSA adsorption of the higher-concentration PCL films. According to the electrochemical measurement results shown in Figure 4, the 316L SS coated with PCL films all exhibited superior corrosion resistance relative to that of the bare 316L SS. This feature arises from the excellent insulating nature of the PCL films. Therefore, PCL films are suitable for the surface modification of 316L SS because they provide good anticoagulant properties and corrosion resistance. Compared to the other PCL films of higher concentrations, the 2% PCL film exhibits a relatively smoother surface morphology, which corresponds to a lower BSA protein adsorption level.

## 5. Conclusions

The surface and protein adsorption properties of 316L SS modified by coatings of PCL films were investigated. The main results and conclusions were as follows:

(1) Water contact angle measurement results showed that the wettability of the PCL films was comparable to that of bare 316L SS because the rough surface morphology of PCL counteracted the hydrophobicity.
(2) BCA protein assay results showed that the 316L SS modified by PCL films all possessed lower adhered BSA concentrations than that of bare 316L SS, suggesting that the anticoagulant properties of the 316L SS surface could be improved by modification with PCL films. The BSA protein adsorption of the PCL films is slightly increased from the 2% to 4% PCL films because the higher-concentration PCL films exhibit rougher surfaces and more hydrogen bonding interactions between the carbonyl functional groups of the PCL films and the carboxylic acid of the BSA.
(3) Electrochemical tests revealed that the corrosion resistance of the 316L SS was effectively enhanced after coating with PCL films, which is favorable to prevent the undesirable metal ions from leaching from stainless steel.
(4) PCL films possessed excellent corrosion resistance and good anticoagulant properties and showed promise for use in biomedical applications, particularly for the surface modification of implants.

## Figures and Tables

**Table 1:** The average *E*_corr_ and *i*_corr_ values determined according to the cathodic and anodic polarization Tafel curves in Figure 3.

Specimens	*E*_corr_ (mV)	*i*_corr_ (nA/cm^2^)
Bare 316L SS	−196.2	97.5
2% PCL	−129.6	84.7
3% PCL	96.0	0.1
4% PCL	84.9	0.3
